# Expert-based collaborative analysis of the situation and prospects of biomarker test implementation in oncology in Spain

**DOI:** 10.1007/s12094-023-03338-8

**Published:** 2024-01-11

**Authors:** Jorge Mestre-Ferrándiz, Blanca Franch Camino, Álvaro Hidalgo, Alicia del Llano Núñez-Cortés, Juan Ernesto del Llano Señarís, Blanca Lumbreras, David Beas Pedraza, Roberto Nuño-Solinís, Luis Paz-Ares, Santiago Ramón y Cajal, Miguel Javier Rodríguez

**Affiliations:** 1 Universidad Carlos III, Madrid, Spain; 2 Fundación Gaspar Casal, Madrid, Spain; 3 Fundación Weber, Madrid, Spain; 4 Universidad Miguel Hernández (UMH), Elche, Spain; 5 Janssen España, Madrid, Spain; 6 AES, Madrid, Spain; 7 Hospital Universitario 12 Octubre, Madrid, Spain; 8 Hospital de Vall d’Hebron, Barcelona, Spain; 9 Hospital Universitario Central de Asturias, Oviedo, Spain

**Keywords:** Oncology, Precision Medicine, Biomarkers, Policy Roadmap, Spain

## Abstract

**Purpose:**

Biomarkers as screening for precision medicine is a fundamental step. The purpose of this article is twofold. First, to highlight the existing barriers in the implementation of Precision Medicine in Spain, with a special emphasis on barriers in access to the determination of biomarkers. Second, to provide a Roadmap that can help implement Precision Medicine equitably at the national level and optimize the use of biomarkers.

**Methods:**

A systematic review of literature (SRL) and a focus group (FG) with multidisciplinary experts has been carried out in 2023. Participants were contacted individually, and discourse analysis was processed anonymously.

**Results:**

We carried out a quantitative (SRL) and a qualitative approach (FG). The discourse analysis and roadmap were sent individually to each expert for approval.

**Conclusions:**

The potential of Precision Medicine has not been fulfilled in Spain. While several regional initiatives are in place, a national plan or strategy around Precision Medicine and use of biomarkers is lacking. In a general context of rapid progress at a global and European level, including the 2021 Europe’s Beating Cancer Plan, it is time to define and implement a National Plan to make the promise come true. While some comparable countries within Europe – such as the UK or France – are mature enough to adopt such strategies, in Spain there is still a long way to go. We consider that the different strands of work outlined in the Roadmap can be used as basis for such purpose.

## Introduction

Currently, the term “Precision Medicine” is widely used; however, understanding its implications for healthcare systems requires further reflection and analysis. The origin of this concept started with the culmination of the Human Genome Project in 2001, and the subsequent widespread sequencing of the human genome, given that Precision Medicine is largely based on genetic information. Diagnostic tests that allow the determination of biomarkers (or biological markers) are positioned as a key tool, and even the cornerstone of Precision Medicine. They allow better results for the patient with a more appropriate use of the limited healthcare resources available, including pharmacological treatments.

The advancement of Precision Medicine is presented as an opportunity to give a significant boost (“leapfrog”) in the prevention, identification, treatment, and follow-up of patients, in particular patients diagnosed with cancer associated to available treatments. There are many definitions of Precision Medicine, sometimes confusing, but the most widespread is possibly this one from US’ National Institutes of Health (NIH) in the USA: “an emerging approach for the prevention, diagnosis and treatment of disease that takes into account the individual, environmental and lifestyle variability of each person” [1]. The new approach to healthcare proposed by Precision Medicine is possible—thanks to advances in genetic knowledge of diseases, technological advances in data analysis, and development of targeted therapies with more effective treatment. In brief, Precision Medicine allows the personalization of healthcare, and the most adequate pharmacological treatment to each patient, resulting in better health outcomes.

The effective use of the determination of biomarkers is a key tool of Precision Medicine. The NIH established the definition of a biomarker as those biological, biochemical, anthropometric, physiological characteristics, etc., objectively measurable, capable of identifying physiological or pathological processes, or a pharmacological response to a therapeutic intervention [2]. In the field of oncology, the identification of a biomarker associated with a specific type of cancer is key since it allows for more precise diagnosis and more effective therapy. Certain tumors, such as lung cancer, have benefitted from a large number of molecular targets and pharmacological treatments associated to these markers, with corresponding increases in survival. More recently, new tumor-agnostic treatments have been developed and authorized, reinforcing the need to develop the concept of Precision Medicine to enable their implementation and uptake.

However, some of the main difficulties associated with the development of precision medicine are the high cost associated with it (even though its cost-effectiveness has been proven in several cancers [3]), the lack of knowledge in the interpretation of genetic and health data, access and availability of genetic tests and errors in the validation of these genetic tests, among others.

With this context, the purpose of this paper is twofold:

First, to highlight the existing barriers in the implementation of Precision Medicine in Spain, with a special emphasis on the barriers in access to the determination of biomarkers.

Second, to offer a Roadmap that can help implement Precision Medicine in a generalized and equitable manner at the national level, with an optimal use of biomarker determination.

## Methods

To achieve the two objectives, two activities were carried out:

1. A scoping literature review using different combinations of the two key search terms/phrases (definitions of precision medicine/biomarkers, and barriers in the use and access of biomarkers in oncology), in PubMed and Google/Google Scholar.
2. A hybrid process of Expert-based Cooperative Analysis [4] of the situation and perspectives of the use of biomarker tests in Spain through a Focus Group with seven experts (Table 1 lists them, with their areas of knowledge). A face-to-face meeting was organized in Madrid on the 16 February 2022, structured in two parts. In the first part, the experts made a diagnosis of the current situation in Spain. Each expert presented their point of views (without reply) on two topics: (a) barriers to the access and use of the determination of biomarkers and the development of Precision Medicine in oncology, and (b) windows of opportunity to promote Precision Medicine in oncology. In the second part, each expert shared their vision (different but complementary and interdisciplinary), to generate a Roadmap that could allow the acceleration of progress in the use of biomarker determination. This could be considered as a driver of Precision Medicine in oncology in Spain.

**Table 1:** Members of the Focus Group

Knowledge area	Name of the expert
Health Economics	Álvaro Hidalgo
Epidemiology	Blanca Lumbreras
Pathology	Santiago Ramón y Cajal
Oncology	Luis Paz-Ares
Regulation	César Hernández
Pharmaceutical industry	David Beas
Policy maker	Miguel Javier Rodríguez
Coordinator	Juan del Llano

## Results

We have structured the main findings in three sections. Overall, we found general consistency across the main topics and barriers identified by the literature review and the experts’ opinions.

### Current situation in Spain

Several reports have been recently published offering a view on access to both biomarker determinations and treatments associated with these determinations in oncology. In 2020, the Spanish Society of Medical Oncology (SEOM) [5], updated a previous study from 2015, analyzing the availability of 11 approved medicine/indication binomia and 5 determinations of biomarkers predictive of response, for the treatment of non-small cell lung cancer, breast cancer, melanoma and ovarian cancer. The study concluded that there was “no standardized procedure or specific regulatory framework for the evaluation, implementation, and financing of diagnostic tests in clinical practice and the average response time from the request for determination of the biomarkers/indications included to the availability of the result varies significantly between biomarkers, Autonomous Communities, and hospitals”.

More recently, another study [6] analyzed access to Next-Generation Sequencing (NGS) and determination of biomarkers in the National Health System (NHS), stressing a low implementation of NGS on a routine basis in Pathology Departments. Similarly, another report [7] assessed, among other aspects, the degree of the implementation (or progress) of Precision Medicine in Spain at a regional level, showing Andalusia, Castilla y León, Catalonia, Galicia, and the Basque Country as the most advanced regions (5 out of the 17 Spanish Autonomous Regions).

Some regions also have their own plans, with different names, that are working well [7], like those of Catalonia and Cantabria. However, these initiatives remain at regional level, and hence are usually fragmented and lack interoperability. In part to resolve this issue at national level, the Institute of Health Carlos III (ISCIII), the national and international reference in biomedical research and public health in Spain, has created the Personalized Medicine Infrastructure associated with Science and Technology (IMPaCT). Its key objective being to mobilize data and generate resources in the cloud to work collaboratively in biomedicine.

Importantly, however, there is still no National Plan for Precision Medicine in Spain. The experts of the Focus Group remarked the need to develop and implement one. Within such a plan, the experts highlighted the need to define a national portfolio of minimum precision medicines tests available. The good news is, since the Focal Group, there have been announcements made by the Spanish Government to fund biomarker determination within the Spanish NHS [6].

### Barriers in access to the determination of biomarkers

Access to the determination of biomarkers is unequal, between countries and, within Spain, between Autonomous Communities. This leads to a paradox, highlighted by Mateo and colleagues [8], and which was corroborated in the Focus Group: if patients do not have access to these diagnostic tests, candidates to a particular therapy cannot be identified, and therefore will not have access to treatments that are linked to these genetic determinations.

Several barriers have been identified in the access and use of diagnostic tests for the determination of biomarkers in clinical practice in Spain. We have grouped them into the following six thematic areas:

#### Public funding for Precision Medicine and the determination of biomarkers

One of the main barriers is the lack of specific funding for it. The determination of biomarkers and the determination by NGS are not included in the portfolio of common services of the NHS. This implies that currently these determinations are financed through other funds, mostly by the pharmaceutical industry. Both the Focus Group and the reviewed literature emphasize this aspect, including a previous study showing that the determination of some of the biomarkers is funded by different pharmaceutical companies in more than 50% of the hospitals [5]. Moreover, it has recently been estimated that the analysis of molecular diagnosis by NGS versus sequential single testing in metastatic non-small cell lung cancer patients from a Spanish reference centre perspective could be considered a cost-effective strategy [9]. The benefits from NGS relative to the single gene testing include the number of alterations detected, treatment with targeted therapies and clinical trial enrollment.

#### Common regulatory framework for its evaluation, implementation, and financing

The lack of a regulatory framework was identified in the Focus Group as one of the most important barriers in Spain. SEOM has already highlighted the lack of clear approval and decision-making procedures [5], something detected almost two decades ago with the availability of the first treatments for women with HER2+ breast cancer.

In addition, economic evaluation systems do not necessarily recognize all the benefits derived from the use of diagnostic tests or biomarker determination; indeed, many countries with established health technology assessment (HTA) programs lack specific national programs to specifically regulate, evaluate, and provide guidance on diagnostic tests. Moreover, among those countries that do have them, there is a notable variability regarding the methods, processes, scope and degree of implementation, and compliance with standards [10]. Therefore, the price of these biomarker tests is often determined based on their costs, rather than in the cost–benefit balance [11]. However, with a well-designed system, the use of these biomarkers will benefit to all, since they can lead to a better use (or even savings) of resources, by specifying and personalizing healthcare. Usually, there can be an associated pharmaceutical treatment to the biomarker. When this is the case, there may be a problem of attributing costs and benefits between the tests and the treatment associated with the results of this test. Regardless of how they are allocated, it must be considered that their value lies in the combination of both, so it is a joint product [10]. And as argued above, the strategy of using NGS can be cost-effective in Spain [9], but also in other countries like the US [12].

#### Infrastructure

Implementation of Precision Medicine requires an infrastructure, beyond biomarkers, including a sufficient laboratory testing network, storage capacity, analysis, and access to information. In addition, the lack of coordination and organization between the different sections of a health system (laboratory, clinicians, patients, managers, etc.) was highlighted during the Focus Group, with a lack of communication to the patients and responsibilities of the different professionals not clearly defined. The development of Artificial Intelligence will play a relevant role in the access and analysis of relevant health data. Nevertheless, currently its use is limited in Spain, as discussed during the Focus Group.

#### Knowledge and training on the subject

The limited knowledge in professionals and a shortage of laboratory personnel to perform such tests was also identified by the Focus Group as a barrier, which is also related to limited funding. Likewise, the patients’ lack of knowledge about Precision Medicine and biomarkers can prevent these patients from being actively involved in requesting biomarker tests [11].

It is worth mentioning Spain is the only European country without a medical specialty in Clinical Genetics. But beyond clinical genetics, the importance of being able to have new professional profiles, such as mathematicians, physicists, biotechnologists, bioinformaticians, biomedical doctors, biostatisticians, and other professionals and analysts, related to the generation and use of data, was highlighted during the Focus Group; however, bureaucratic obstacles to hiring such professionals in national public organizations were stressed.

#### Political leadership, stakeholder participation and public–private partnerships

Two key aspects were identified by the Focus Group in particular, to support the implementation of Precision Medicine: political leadership that facilitates change and transformation, and the commitment of health professionals to this change (which would imply taking them into consideration in the decisions so they can also lead the initiatives). There was consensus among the experts that this leadership does not currently exist at the national level.

Moreover, greater public–private collaboration is currently needed, with a much longer horizon, as well as greater strategic planning, to implement and develop Precision Medicine.

#### Problems associated with the characteristics of current biomarkers

First, there are problems with the evidence generated at the various stages in biomarker development. Most of the available evidence is focused on the research in the initial phases of the development of biomarkers (to demonstrate pre-analytical and analytical validity). This implies that there are fewer studies in the more advanced phases that aim to generate evidence on clinical validity, to make the leap to the application in the patient and generate evidence of clinical utility.

Assessing pre-analytical and analytical validity also has associated problems, which were detailed during the Focus Group. On the one hand, there is variability in sample handling protocol for pre-analysis (for example, defining the type of biological sample, the procedure and time to be collected, processed, stored). To solve this problem, recommendations have already been published for the standardization of all pre- and analytical sample procedures [13].

Perhaps, one of the most important barriers related to current biomarkers is the lack of studies on their clinical utility (that is, on effectiveness or benefit–risk ratio, both in absolute terms and relative to other existing techniques), something that was clearly reflected during the Focus Group. In addition, there was consensus among the experts that it is precisely in the implementation in clinical practice where the great problem lies. Moreover, a study has shown that only a small proportion of biomarkers initially presented for authorization were evaluated and considered clinically useful [10].

Second, there is a limited number of treatments associated with biomarkers, although for oncohematology specifically, and as commented during the Focus Group, 30% of the medicines approved by the European Medicine Agency (EMA) between 2015 and 2020 are associated with biomarkers [14]. However, one of the barriers to the development and use of biomarkers is that many companies fail to integrate a biomarker strategy early in the drug development program, which could avoid, or at least minimize, the challenges that the biomarker faces at market launch [15].

Third, the cost of performing tests to identify these biomarkers in patients is often not affordable for many health centers—mainly due to a lack of economies of scale (i.e., volumes are not sufficiently high to reduce the average costs of doing the test).

Fourth, technology now enables NGS tests capable of assessing several biomarkers at once, faster, and more efficiently, compared to the more traditional situation of assessing just one gene/biomarker. However, in clinical practice today, tests for a single gene continue to be performed inconsistently and without optimizing patient care, resulting in a situation where neither are used optimally.

Fifth, and as identified by SEOM, the time elapsed to obtain the results acts as a barrier, although in many cases, this delay has been related to the outsourcing of the test and not to problems directly associated with it [5].

Finally, the importance of quality controls to ensure high-quality tests was also raised during the Focus Group and identified previously [11] as a factor determining access to such tests, since there can be up to 10 or 20% false positives and negatives, depending on tumor and patient’s characteristics.

### Roadmap

We propose the implementation of a “National Plan for Precision Medicine” governed and executed by public–private partnerships (PPP). This partnership should include: representatives of autonomous regions, clinician associations, private and public laboratories where tests are run, companion diagnostics companies, biomarker developers, pharmaceutical companies and specialists related to Data Science.

#### Challenge 1: ensure an agile legal framework for innovation (i.e., for biomarker development)

Enactment of a common legal framework that ensures equal access to biomarkers across Spain, independent of the Autonomous Region.

Inclusion of biomarkers within the public healthcare services, as part of a national portfolio of minimum precision medicine tests available to patients across all regions.

Definition of the legal framework to regulate the use of Artificial Intelligence models for clinical decision-making.

To lead this effort, we propose a mutually beneficial collaboration between leading public organizations and private entities, especially those specialized in areas where the public system is less mature.

A joint organization could pool both public and private data and expertise (health economists, lab technicians, Big Data Analysts…) for two missions: (1) provide expert input for the legal framework – focusing on the AI regulation, where Data & Tech ‘outside’ expertise is needed; and (2) run more effective, faster studies to determine the national biomarker portfolio. This organization could find inspiration in systems such as the US Biomarkers Consortium (FNIH).

#### Challenge 2: generate a financing and operating model to ensure equal access to biomarkers

To make an efficient use of the health system resources is key to use diagnostic tests designed to detect most actionable biomarkers simultaneously. Therefore, we recommend a financial model for biomarkers based on the unmet need and the access of medical products indicated and reimbursed for those patients harboring the specific mutation.

Define a financing model of biomarkers, including innovative-based pricing schemes.

Review of current identification of referenced patients, to ensure correct reimbursements between centers.

Selection of public and private reference biomarkers centers distributed across the different Autonomous Regions in nodes (e.g., following the French model as starting point); and where reference centers can mentor other centers not included.

Special incentives to bolster access equality for reference centers in Autonomous Regions with higher inequalities.

Outsourcing of infrastructure to private partners wherever it can create savings for the public sector and creation of a public–private collaboration consortium.

#### Challenge 3: ensure the plan’s viability, in terms of people and technology.

In the current “moment of change” (IMPaCT, EU next-gen funds, 1 million Genome, Spanish PERTE [16], etc.), we propose several measures to speed up transformation:

Integrated public–private organization model, led by a public central entity (including regulators) along with private and public advisors for some areas (e.g., AI), and together with the regions. 

Re-skilling of public entities to include new skills and inclusion of new profiles key for the mid/long term, including statisticians, mathematicians, and data scientists.

Ensuring a new technological infrastructure to operate the model, using synergies with other digitization initiatives.

## Conclusions

The promise of Precision Medicine has been present for over 20 years; however, in Spain, this promise has not been fulfilled. While several regional initiatives are in place, a national plan or strategy around Precision Medicine and use of biomarkers is lacking. In a general context of impulse at global and European level, including the 2021 Europe’s Beating Cancer Plan [17], it is time to define and implement a National Plan to make the promise come true. Some comparable countries within Europe—such as the UK or France [18], are mature enough to adopt such strategies, while in Spain there is still a way to go. We hope the different strands of work outlined in the Roadmap can be used as basis for such purpose, with the objective of ensuring equitable access to Precision Medicine, including the targeted therapies, in Spain.

## Data Availability

Not applicable for this paper.
